# Flexural Exanthema From Enfortumab Vedotin

**DOI:** 10.7759/cureus.8102

**Published:** 2020-05-13

**Authors:** Dinesh Keerty, Laura Graham, Elizabeth Haynes, Timothy N Hembree

**Affiliations:** 1 Internal and Hospital Medicine, H. Lee Moffitt Cancer Center and Research Institute, Tampa, USA; 2 Internal and Hospital Medicine, Moffitt Cancer Center, Tampa, USA

**Keywords:** adverse drug reaction, urothelial malignancy, drug rash

## Abstract

Urothelial malignancies are commonly treated with platinum-based therapies. Newer trials have tested antimitotic therapies such as enfortumab vedotin as viable treatment therapy for refractory malignany. Enfortumab vedotin targets nectin-4, a member of a family of calcium-dependent, immunoglobulin-like adhesion molecules found in adherens junctions and expressed in various epithelial malignancies, including bladder, breast, lung, ovarian, head/neck, and esophageal cancers. We present a case of a patient with symmetrical drug-related intertriginous and flexural exanthema secondary to enfortumab. He was successfully treated with topical corticosteroids. Cutaneous toxicity appears to be a common adverse reaction in this growing class of antibody-drug conjugates.

## Introduction

Enfortumab vedotin is an antimitotic antibody-drug conjugate that inhibits microtubule assembly [1]. It is currently approved to be utilized in urothelial carcinomas, ovarian cancers, and non-small cell lung cancers [2] . Common toxicities that have been attributed to enfortumab have been fatigue, peripheral neuropathy, skin rashes, gastrointestinal issues, and hematological suppression [3]. We present a case of a patient with symmetrical drug-related intertriginous and flexural exanthema secondary to enfortumab.

## Case presentation

**Figure 1:**
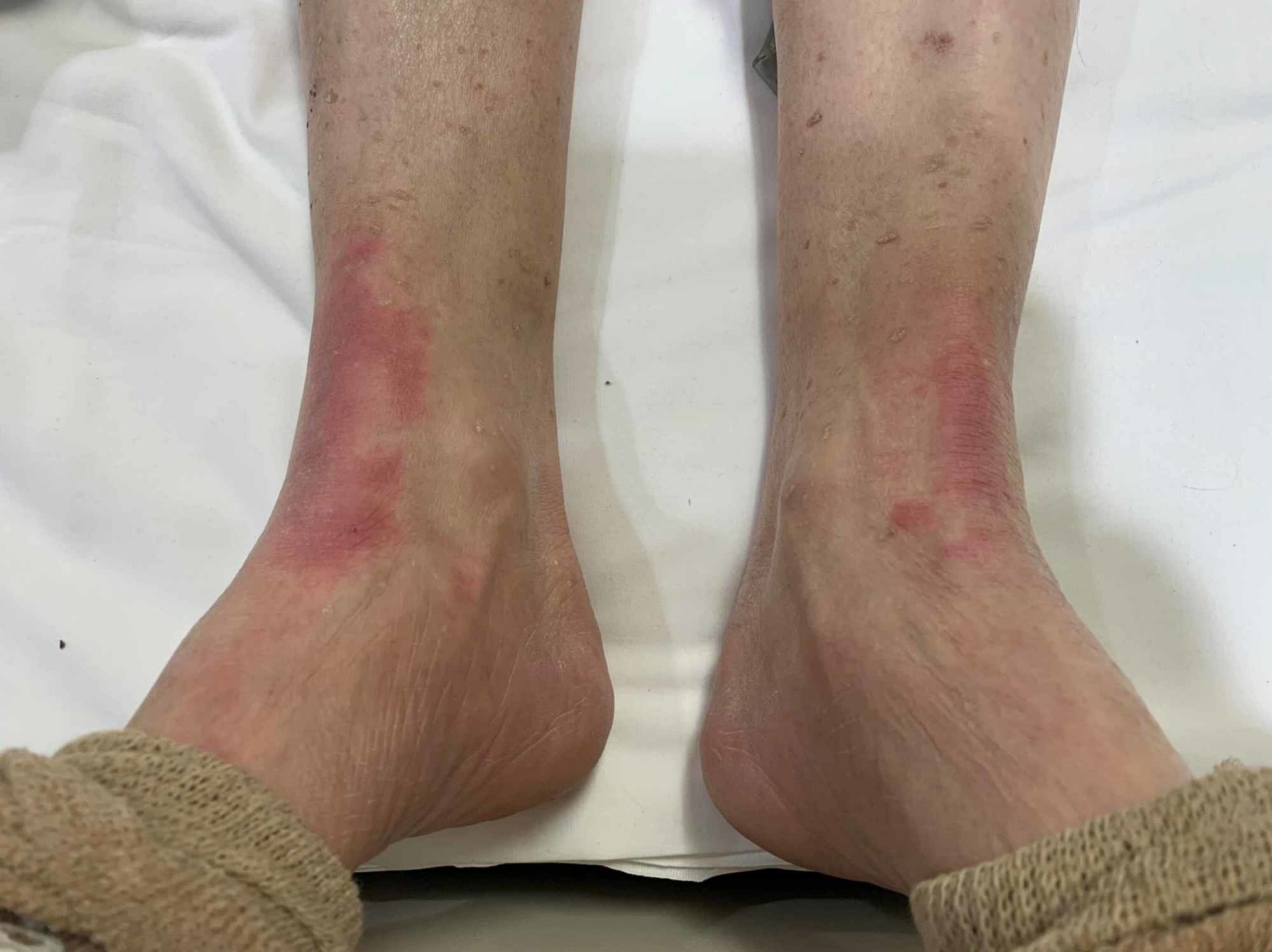
Bilateral flexural exanthema of feet

A 64-year-old male with metastatic urothelial cancer presented to the emergency department with complaints of multiple areas of swollen, erythematous patches in bilateral armpits, groin regions, elbow folds, and dorsal aspects of feet. The patient was started on a new treatment with enfortumab vedotin about one month ago. He received a total of five doses with the last treatment received five days back. He denied any fevers, chills, nausea, vomiting, or diarrhea. He stated that the erythematous patches started two days ago, sudden in onset, in his right axillary region and by the end of the day it had appeared in all the other sites (Figure 1). 

The erythematous patches started swelling and caused him burning pain. The patient has baseline peripheral neuropathy from previous carboplatin-induced toxicity. The patient’s labs showed a white count of 9,820 cells/uL, platelet count of 203 K/uL, and a normal comprehensive metabolic panel. A procalcitonin and lactic acid were procured which were negative for active infection. The patient was started on diphenhydramine and triamcinolone 0.1% cream. Over the span of seven days, he soon started feeling relief and the rash dissipated. His oncologist noted significant improvement of the urothelial cancer with enfortumab treatment. Since the patient had resolution of the rash, enfortumab was resumed at a 20% dose reduction for a span of three weeks. Over the three-week period, he did not have recurrence of the flexural exanthems. 

## Discussion

Nectin-4 arises from members of calcium-dependent immunoglobulin adhesion molecules located in adherens junctions. They are expressed in various epithelial cancers such as bladder, breast, lung, ovarian, oropharyngeal, and esophageal cancers. Enfortumab vedotin is designed to act on nectin-4 to disrupt the mitotic process [1]. Phase 1 data for enfortumab vedotin in the treatment of metastatic urothelialcarcinoma have promising results, but have noted treatment-related adverse events like rash, nausea, and decreased appetite [4]. Skin reactions, such as symmetrical drug-related intertriginous and flexural exanthemas, constitute a grade 3 or grade 4 reaction. Data have shown that these reactions can occur in 52%-54% of cases of patients on the medication, but they do not delineate duration prior to reaction. Some can progress to bullous dermatitis, exfoliative dermatitis, and/or palmar-plantar erythrodysesthesia. The median time to onset of skin reactions has been estimated to be one month. Of patients who experienced rash, nearly two-thirds experienced complete resolution and approximately one-fifth experienced partial improvement [3]. As per guidelines, topical corticosteroids and antihistamine usage has been warranted. Also, withholding of the medication till the symptoms resolution was recommended.

## Conclusions

Enfortumab vedotin is a newer antimitotic agent being used to treat urothelial malignancies. As in case with other chemotherapeutic agents, dermatological side effects can arise. This case elucidates potential flexural exanthemas that can result from the medication. These reactions should be treated by steroids and withholding of enfortumab vedotin. Re-initiation of treatment should be done with careful monitoring as these benign exanthemas can progress to more complex issues such as Stevens-Johnson syndrome. 
